# Embryo CHH hypermethylation is mediated by RdDM and is autonomously directed in *Brassica rapa*

**DOI:** 10.1186/s13059-021-02358-3

**Published:** 2021-05-06

**Authors:** Tania Chakraborty, Timmy Kendall, Jeffrey W. Grover, Rebecca A. Mosher

**Affiliations:** 1 The School of Plant Sciences, The University of Arizona, Tucson, AZ 85721 USA; 2 Department of Molecular and Cellular Biology, The University of Arizona, Tucson, AZ 85721 USA

**Keywords:** RNA-directed DNA methylation, DNA methylation, CHH methylation, Embryo development

## Abstract

**Background:**

RNA-directed DNA methylation (RdDM) initiates cytosine methylation in all contexts and maintains asymmetric CHH methylation. Mature plant embryos show one of the highest levels of CHH methylation, and it has been suggested that RdDM is responsible for this hypermethylation. Because loss of RdDM in *Brassica rapa* causes seed abortion, embryo methylation might play a role in seed development. RdDM is required in the maternal sporophyte, suggesting that small RNAs from the maternal sporophyte might translocate to the developing embryo, triggering DNA methylation that prevents seed abortion. This raises the question of whether embryo hypermethylation is autonomously regulated by the embryo itself or influenced by the maternal sporophyte.

**Results:**

Here, we demonstrate that *B. rapa* embryos are hypermethylated in both euchromatin and heterochromatin and that this process requires RdDM. Contrary to the current models, *B. rapa* embryo hypermethylation is not correlated with demethylation of the endosperm. We also show that maternal somatic RdDM is not sufficient for global embryo hypermethylation, and we find no compelling evidence for maternal somatic influence over embryo methylation at any locus. Decoupling of maternal and zygotic RdDM leads to successful seed development despite the loss of embryo CHH hypermethylation.

**Conclusions:**

We conclude that embryo CHH hypermethylation is conserved, autonomously controlled, and not required for embryo development. Furthermore, maternal somatic RdDM, while required for seed development, does not directly influence embryo methylation patterns.

**Supplementary Information:**

The online version contains supplementary material available at 10.1186/s13059-021-02358-3.

## Background

DNA methylation is an epigenetic modification that can modulate chromatin structure and gene expression [1]. Plants methylate cytosines in all sequence contexts (CG, CHG, and CHH, where H is any base other than G) and use specific methyltransferases to maintain each context after replication [2]. In addition, the RNA-directed DNA methylation (RdDM) pathway is responsible for de novo methylation, a process that is most clearly observed at CHH positions [3]. RdDM functions primarily at the edges of euchromatin transposons, where constant re-establishment of methylation might be necessary [4, 5].

RdDM can be divided into siRNA production and DNA methylation stages. During siRNA production, RNA polymerase Pol IV produces single-stranded RNA transcripts which are copied into double-stranded RNA by RNA-DEPENDENT RNA POLYMERASE 2 (RDR2) and cut into 24-nucleotide small interfering (si)RNAs by DICER LIKE 3 (DCL3) [6–8]. To mediate DNA methylation, these 24-nt siRNAs are loaded onto ARGONAUTE 4 (AGO4), which interacts with a non-coding scaffold transcript produced by RNA polymerase V and recruits DOMAINS REARRANGED METHYLTRANSFERASE 2 (DRM2) to institute methylation marks on cytosine bases [9–11]. These two stages of RdDM frequently occur in cis but can also function in trans due to siRNA-AGO4 loading in the cytoplasm [12]. siRNAs can act in trans to trigger DNA methylation at allelic sites [13, 14] or at homologous non-allelic sites [15] or might move between cells to act non-cell autonomously [16].

With the exception of *Arabidopsis*, which has only a small reduction in seed size, loss of RdDM in most species results in disruption of reproductive development, indicating that RdDM is necessary for successful sexual reproduction [17–21]. Mature embryos accumulate high levels of CHH methylation in *Arabidopsis*, soybean, and chickpea, suggesting that RdDM might enable reproduction through hypermethylation of the mature embryo [22–27]. In *Arabidopsis*, the developing endosperm is demethylated at sequences that show hypermethylation in the embryo, leading to the hypothesis that siRNAs produced in the endosperm might move to the embryo to direct methylation [22, 28–30]. The movement of siRNAs between the maternal integuments and the filial tissues has also been proposed [31]. However, embryos produced through somatic embryogenesis also display hypermethylation, despite a lack of association with either endosperm or maternal integuments [27] and torpedo-stage *Arabidopsis* embryos accumulate roughly equal maternal and paternal siRNAs [32].

Here, we show that *Brassica rapa* mature embryos are hypermethylated in the CHH context in both euchromatin and heterochromatin, and we demonstrate that this process requires RdDM. Although maternal RdDM is required for seed development, it is not sufficient for embryo hypermethylation, and methylation in the CHH context is not necessary for proper seed development. Furthermore, we find no evidence that hypermethylation of the embryo is driven by siRNAs produced in adjacent tissues, suggesting that embryo CHH hypermethylation is entirely autonomous.

## Results

### *Brassica rapa* embryos are hypermethylated in the CHH context

**Fig. 1:**
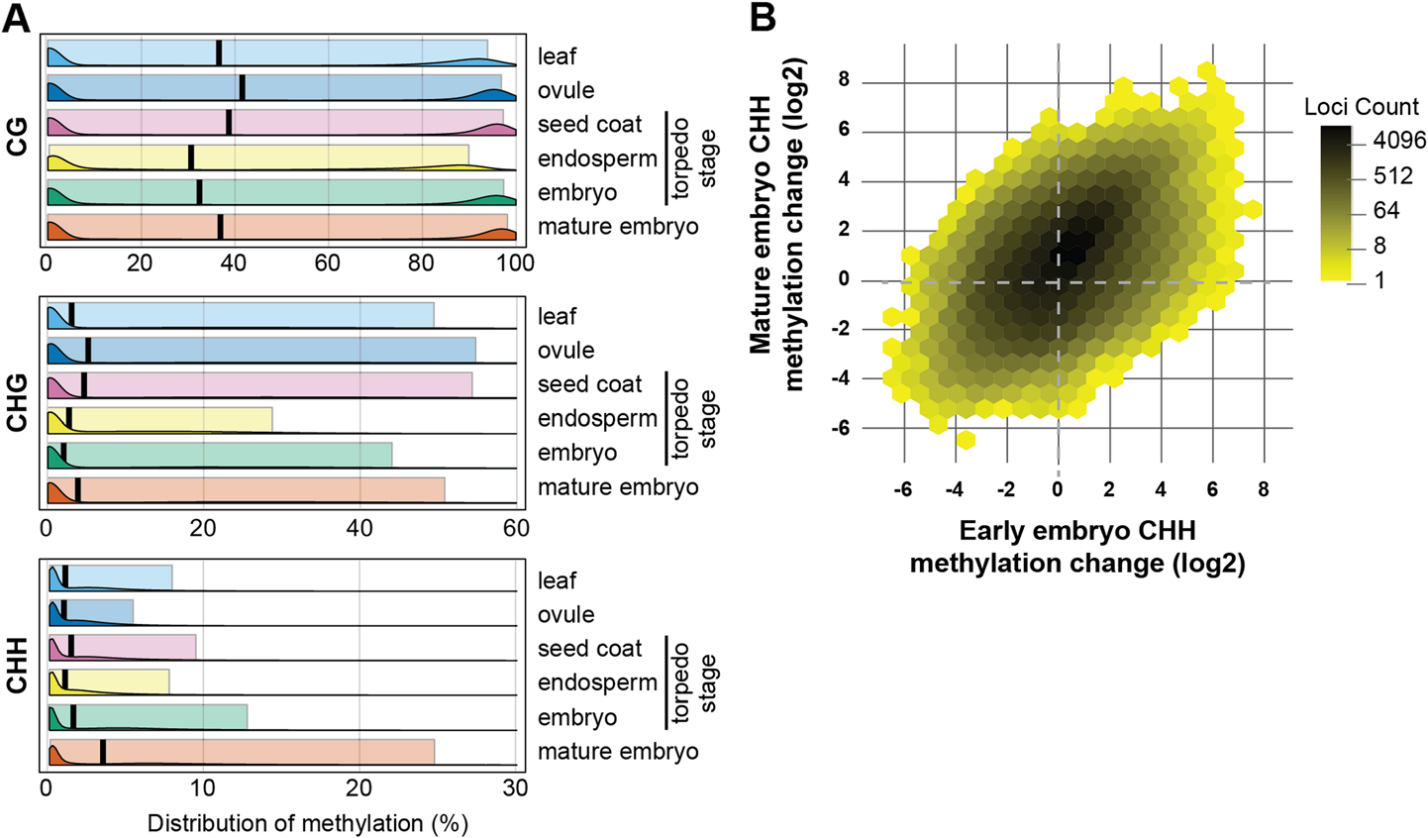
*B. rapa* embryos are hypermethylated at CHH sites. **a** Distribution of methylation levels in 300-nt windows across the *B. rapa* genome. Ridge plots display the density of average methylation in each context, while background box plots enclose the 10th to 90th percentiles of the data. The black bar marks the median for each tissue/context combination. Only windows with a read depth ≥ 5 over all cytosines were included (approximately 1 million windows per tissue/context combination). **b** Increased CHH methylation (log_2_ fold change relative to leaves) is correlated in torpedo and mature embryos; 752,405 300-nt windows with a read depth of at least 5 in both tissues are plotted

To analyze global methylation levels in mature embryos, we performed whole-genome bisulfite sequencing on embryos dissected from dry seeds and compared the resulting data with other reproductive tissues (ovule, endosperm, and early embryo) and a non-reproductive control (leaf). Bisulfite conversion was greater than 99% in all samples, with a read depth coverage of > 9 (Additional file 1: Fig. S1). We calculated methylation in 300-bp non-overlapping windows for all three sequence contexts (Fig. 1a). CG methylation was largely unchanged across the tissues, with the exception of endosperm, which was demethylated in CG and CHG contexts, consistent with the observations in *Arabidopsis* and rice [33–35]. As in *Arabidopsis*, soybean, and chickpea [22–27], we also observed elevated global CHH methylation in *B. rapa* mature embryos, with moderately increased CHH methylation in torpedo-stage embryos (Fig. 1a and Additional file 1: Fig. S1). The increased CHH methylation in torpedo-stage embryos was correlated with CHH hypermethylation in mature embryos (Fig. 1b, correlation coefficient = 0.6), indicating that hypermethylation is a gradual process during embryogenesis.

**Fig. 2:**
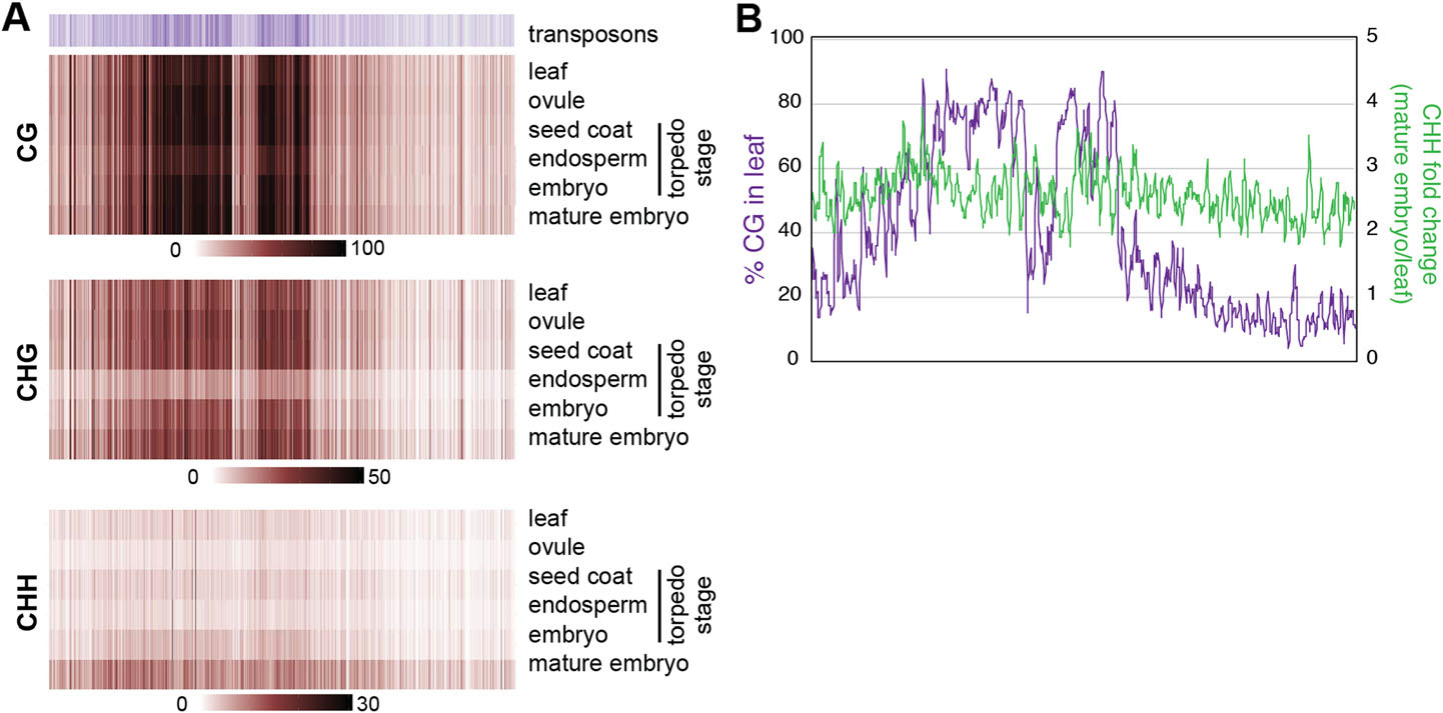
Embryo CHH hypermethylation is not restricted to the pericentromere. **a** Heatmaps of transposon density or methylation level in 25-kb windows across chromosome 10. Each methylation context has its own scale bar to visualize changes across tissues. Other chromosomes are presented in Additional file 1: Fig. S2. **b** CHH hypermethylation in mature embryos (green line) is not correlated with the amount of CG methylation in leaves (purple line). Five-window rolling average of 25-kb windows across chromosome 10 are plotted

To assess the types of chromatin responsible for embryo hypermethylation, we analyzed methylation levels in 25-kb windows across each chromosome (Fig. 2a and Additional file 1: Fig. S2). Pericentromeric heterochromatin, which has a denser accumulation of transposons, was strongly methylated in CG and CHG contexts in all tissues. The sole exception was endosperm, which as expected showed a small reduction in CG methylation and stronger loss of CHG methylation. In comparison with these heterochromatic marks, CHH methylation was distributed more equally across the length of the chromosome. Increased CHH methylation in mature embryos relative to other tissues was readily apparent. In *Arabidopsis*, embryo hypermethylation occurred primarily in pericentromeric heterochromatin [22, 23], while in soybean somatic embryos, CHH hypermethylation was seen across the genome [27]. We observed a similar degree of CHH hypermethylation in both the CG-dense pericentromeric regions and the chromosome arms (Fig. 2b), indicating that both heterochromatin and euchromatin are targets of CHH methylation during *B. rapa* embryo development.

**Fig. 3:**
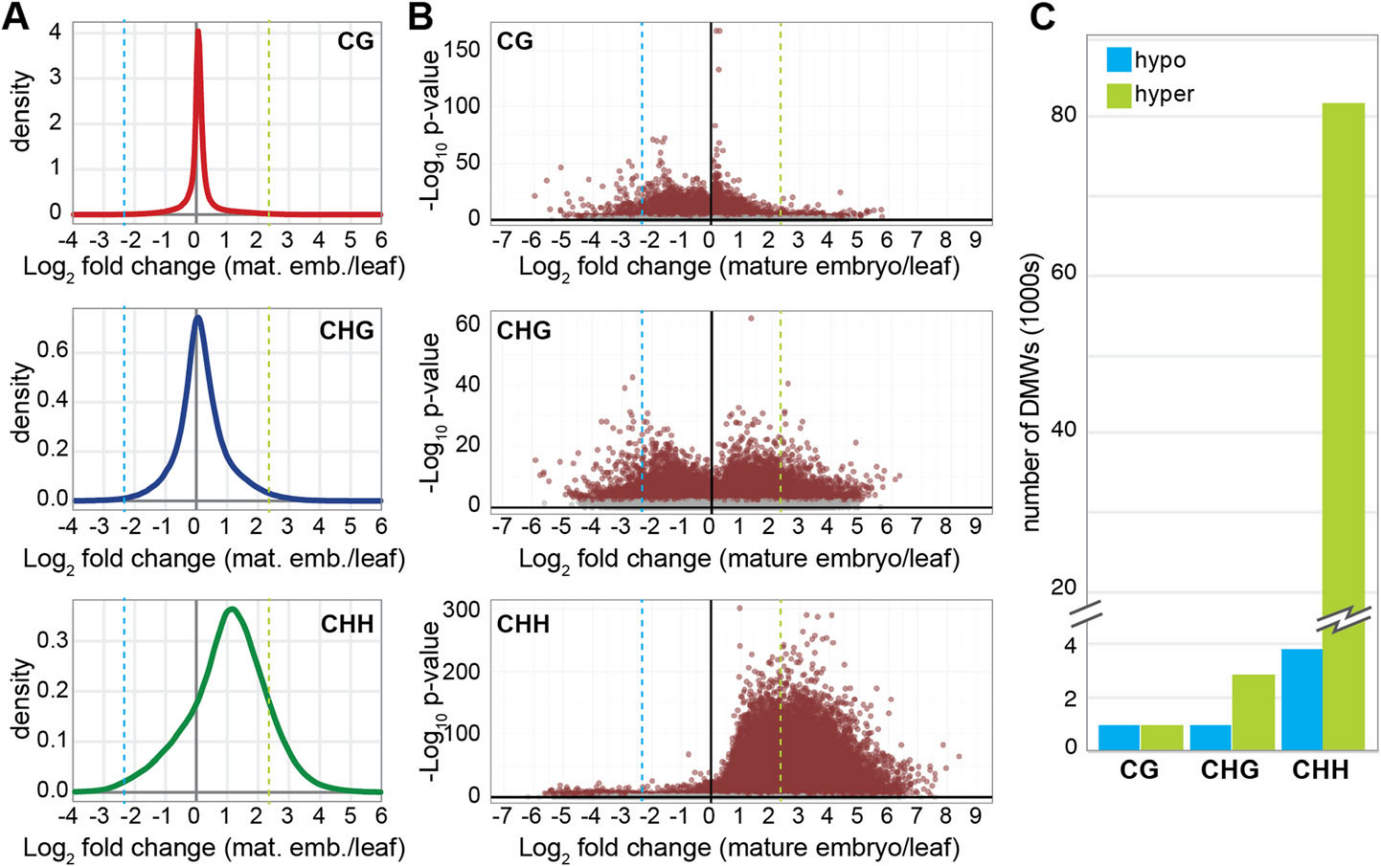
Identification of embryo differentially methylated windows. Density distributions (**a**) and volcano plots (**b**) of methylation fold change in mature embryos versus leaf for three cytosine contexts; 300-nt windows with a read depth of at least 5 are plotted (650,665 CG, 686,741 CHG, and 869,526 CHH windows). The dashed blue line marks 5-fold hypomethylation, and the dashed green line marks 5-fold hypermethylation. Windows above this threshold with an FDR-adjusted *p* value <0.005 were collected for subsequent DMW analysis. **c** Number of differentially methylated windows (DMWs) passing the above thresholds in each methylation context

Finally, we assessed embryo methylation relative to leaf for each cytosine context within each 300-nt window. Most windows were unchanged with respect to CG and CHG methylation, while CHH methylation showed a pronounced shift toward hypermethylation (Fig. 3a). These changes were highly significant (Fig. 3b), but we selected only windows with the strongest changes in methylation for further analysis. We defined differentially methylated windows (DMWs) as those with at least 5-fold (log_2_ = 2.32) increase or decrease in methylation in mature embryo compared to leaf, and an FDR adjusted *p* value less than 0.005 (Fig. 3a, b, Additional file 1: Fig. S3). Hypermethylated windows were more abundant than hypomethylated windows for each sequence context, with CHH hyper-DMWs vastly outnumbering other DMWs in other sequence contexts (Fig. 3c).

Together, our observations demonstrate that mature *B. rapa* embryos are extensively hypermethylated at CHH sites across the genome, and this hypermethylation is the primary difference between the leaf and embryo methylation patterns. This hypermethylation is widespread, not limited to pericentromeric heterochromatin, and progressive throughout embryogenesis.

### Embryo CHH hypermethylation is dependent on RdDM

RdDM is the major pathway for de novo methylation in all sequence contexts, and its activity is frequently observed through the accumulation of CHH methylation. However, most of the CHH methylation in the genome is instead placed by CHROMOMETHYLTRANSFERASE 2 (CMT2) [18, 36]. Kawakatsu and colleagues [23] demonstrated that both RdDM and CMT2 contribute to CHH methylation in the embryo, but their analysis did not determine which process was responsible for the hypermethylation relative to non-embryonic tissues. Small RNA accumulation at hypermethylated regions is correlated with embryo hypermethylation [22, 24, 26], but it is not clear whether siRNA accumulation is required for increased methylation, or whether these two processes occur independently.

**Fig. 4:**
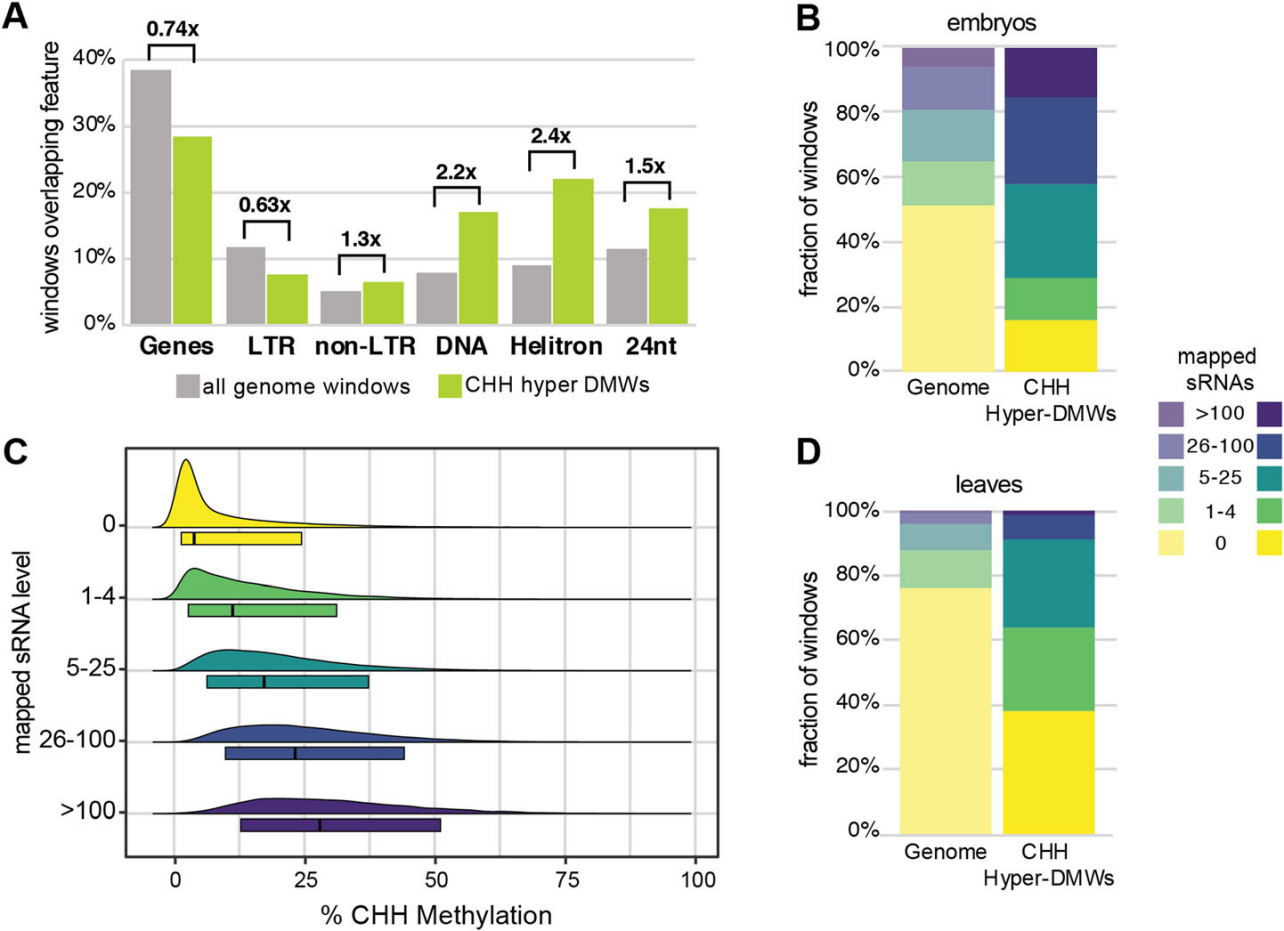
small RNA production is correlated with embryo hypermethylation, but not hypomethylation in leaves. **a** Enrichment or depletion of genomic features at CHH hyper-DMWs. The percentage of CHH hyper-DMWs overlapping annotated genomic features is plotted compared to the percentage of overlap for all genomic windows with comparable read depth. All differences are significant at *p*<2.2e−16. **b**, **d** CHH hyper-DMWs and all genomic windows were binned based on the number of sRNAs mapping to them in torpedo embryos or leaves, and the fraction of windows in each bin is shown. The embryo sRNA library has 39.1 million mapped reads, while the leaf library has 10.8 million reads. **c** Absolute CHH methylation in mature embryos is plotted as a function of the number of mapped sRNAs in torpedo embryos at all CHH hyper-DMWs. Box plots circumscribe the 10th–90th percentiles, and the black bar marks the median

Our analysis also implicates RdDM in embryo CHH hypermethylation. Firstly, compared to all genomic windows with sufficient WGBS read depth, embryo CHH hyper-DMWs are significantly enriched for class II DNA elements and class III Helitrons (Fig. 4a). CHH hyper-DMWs are also significantly depleted at genes and at long terminal repeat (LTR) retroelements, following the characteristic pattern of RdDM loci in *B. rapa* [19]. Most importantly, CHH hyper-DMWs are enriched for loci previously shown to produce 24-nt siRNAs (Fig. 4a).

To further investigate the association between CHH hyper-DMWs and siRNAs, we analyzed siRNA accumulation at CHH hyper-DMWs in torpedo-stage embryos (Additional file 1: Fig. S2). CHH hyper-DMWs have a significantly higher accumulation of siRNAs than the genome as a whole (Fig. 4b), and windows with greater siRNA accumulation in torpedo embryos have higher CHH methylation in mature embryos (Fig. 4c). However, many CHH hyper-DMWs lack substantial siRNA accumulation, a pattern also detected in chickpea [26]. We also observed a similar enrichment of siRNAs at CHH hyper-DMWs in leaves despite the 5-fold or greater difference in methylation between these tissues (Fig. 4d). This suggests that while production of 24-nt siRNAs is associated with embryo hypermethylation, similar 24-nt siRNA accumulation in leaves does not result in similar hypermethylation.

**Fig. 5:**
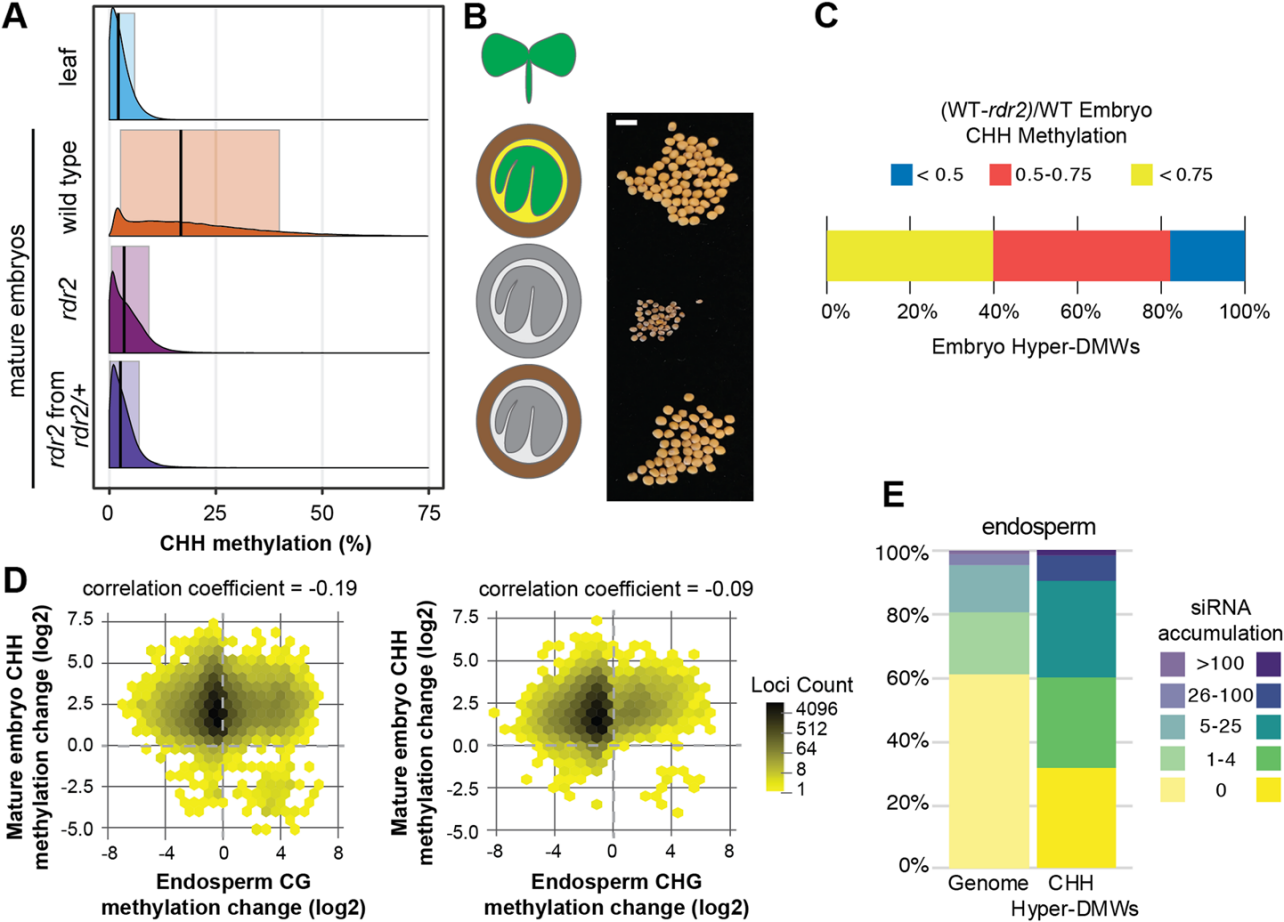
Embryo hypermethylation is determined by filial genotype. **a** Distribution of CHH methylation at CHH hyper-DMWs in leaves and mature embryos. *rdr2* embryos were derived either from *rdr2* homozygous mothers (maroon) or from *rdr2/+* heterozygous mothers (purple). **b** Cartoon and images of representative seeds measured in (**a**). Colored tissues have functional RdDM; gray tissues are deficient in RDR2. Scale bar is 5 mm. **c** Hex plots of mature embryo CHH methylation change by torpedo-stage endosperm CG (left) or CHG (right) methylation. **d** siRNA accumulation in endosperm at CHH hyper-DMWs. The endosperm siRNA library has 19.6 million mapped reads

To directly test whether RdDM is required for embryo hypermethylation in *B. rapa*, we assayed the differences in methylation levels at CHH hyper-DMWs between wild type and an RdDM-deficient mutant, *braA.rdr2-2* (*rdr2* hereafter) (Grover et al. [19]). Mature *rdr2* embryos have a clear reduction in CHH and CHG methylation compared to wild-type embryos (Fig. 5a, b), with CHH methylation levels similar to wild-type leaves. In contrast, CG methylation at these CHH hyper-DMWs is *rdr2*-independent. We also compared WT and *rdr2* methylation at individual loci (Fig. 5c). Over 80% of CHH hyper-DMWs lose at least half of their methylation in the *rdr2* mutant, demonstrating that embryo hypermethylation in the CHH context is primarily due to RdDM, and that other methylation pathways have only a minor contribution to embryo CHH hypermethylation.

### No evidence for endosperm-directed hypermethylation of the embryo

It has been suggested that demethylation of the endosperm allows the production of siRNAs that target DNA methylation in the embryo [22, 28–30]. To determine whether there is an association between endosperm demethylation and embryo hypermethylation, we compared the changes in methylation levels in these tissues. Compared to leaf samples, endosperm is demethylated for both CG and CHG, while embryos are hypermethylated for CHH (Fig. 1a). However, there is no correlation between CG or CHG demethylation in the endosperm and CHH hypermethylation in the embryo, whether we assessed all genomic windows (Fig. 5d) or only the CHH hyper-DMWs (Additional file 1: Fig. S4). We measured correlations between embryo and endosperm methylation in multiple ways, both globally and at CHH hyper-DMWs (Additional file 1: Fig. S3). Absolute CHH methylation in the embryo is positively correlated with all methylation contexts in the endosperm, indicating that the embryo hypermethylated loci tend to have higher absolute methylation in the endosperm. When fold change in methylation relative to leaf samples are measured, only CHH methylation is correlated between these tissues, indicating that regardless of their absolute methylation level, the filial tissues are coordinately increasing CHH methylation. Only when we use the difference in absolute methylation as a metric do we find a correlation between endosperm CG/CHG demethylation and embryo CHH hypermethylation. However, this correlation is not strong and becomes weaker when only the CHH hyper-DMWs are assessed. Together, these results suggest that while similar loci may be demethylated in endosperm and hypermethylated in embryos, there is no evidence that demethylation of endosperm causes hypermethylation of embryos in *B. rapa*.

Because the presumed mechanism whereby endosperm demethylation triggers embryo hypermethylation is transport of siRNAs, we also assessed siRNA production at CHH hyper-DMWs in developing endosperm (Fig. 5e). CHH hyper-DMWs produce more siRNAs than the genome as a whole, but at a level that is comparable to developing embryos or leaves (Fig. 4b, d), suggesting that siRNA production occurs at these windows consistently across tissues and is not a response to endosperm demethylation. On the whole, we find no evidence that demethylation of the endosperm triggers siRNA production to cause hypermethylation of the mature embryo.

### Maternal sporophytic RdDM is not sufficient for embryo hypermethylation

RdDM mutants in *Capsella rubella* and *Brassica rapa* show a high rate of seed abortion that is dependent on the maternal somatic genotype rather than the filial genotype [19, 37]. The few seeds that are produced from *rdr2* plants are smaller and irregular in size and shape (Fig. 5b). Because a functional RdDM pathway in the maternal sporophyte is required for seed development, we hypothesized that maternal sporophytic RdDM might drive hypermethylation of the developing embryo. To test this hypothesis, we pollinated heterozygous (*rdr2/RDR2*) pistils with homozygous (*rdr2/rdr2*) pollen and identified *rdr2* embryos that developed in the presence of functional maternal sporophytic RdDM. Compared to methylation levels of *rdr2* mutant embryos from homozygous mutant mothers (*rdr2/rdr2*), we did not observe the restoration of embryo CHH hypermethylation (Fig. 5a, b).

To further probe the possibility that maternal sporophytically derived siRNAs might trigger hypermethylation of the embryo, we assessed DNA methylation levels at previously defined “siren” loci [31]. These loci produced over 90% of the 24-nt siRNAs in maternal integuments and are also the most highly expressed siRNA loci in the endosperm. We found no evidence that siRNAs produced from siren loci in the integument were able to direct DNA methylation in *rdr2* embryos (Additional file 1: Fig. S5). These results indicate that although siRNA production in the maternal sporophyte is necessary for seed development, it is not sufficient for embryo hypermethylation.

Together, these observations provide no evidence that embryo hypermethylation is directed by siRNAs from another tissue. Combined with the observation that embryos formed through somatic embryogenesis also have elevated DNA methylation despite their lack of interaction with endosperm or integuments, we conclude that embryo hypermethylation is autonomously directed.

## Discussion

Seeds form the majority of the world’s food supply, making the development of the seed and interactions between its multiple tissues critically important areas for research. Double fertilization gives rise to the diploid embryo and the triploid endosperm, which are surrounded by the seed coat, a maternal somatic tissue. Communication between maternal and filial tissues, as well as between the embryo and endosperm, is essential to coordinate the development of a seed [38, 39]. Small RNAs have been proposed to move between seed tissues and to establish robust methylation of transposons at this transition between generations [22, 28–31].

Here, we provide direct evidence that 24-nt siRNAs are responsible for the hypermethylation of mature embryos by demonstrating that *rdr2* embryos lose hypermethylation. However, our evidence suggests that these siRNAs are derived autonomously in the embryo and are not transported from other tissues. Maternal sporophytic *RDR2* (and hence, siRNA production) is not sufficient for embryo hypermethylation (Fig. 5a, b), clearly indicating that the siRNAs responsible for hypermethylation are produced in the filial tissues. Because the embryo and the endosperm have the same genotype (differing only in maternal ploidy), we cannot separate them genetically. However, we find that endosperm does not produce more siRNAs than embryos from CHH hyper-DMWs (Fig. 5e), nor is there a correlation between endosperm CG/CHG demethylation and embryo CHH hypermethylation (Fig. 5d). Furthermore, somatic soybean embryos produced in tissue culture also display embryo hypermethylation [27]. The most parsimonious explanation for these observations is that embryo CHH hypermethylation is autonomously directed by siRNAs synthesized in the embryo.

Although maternal sporophytic siRNA production is not sufficient for embryo hypermethylation, it remains possible that siRNAs from the maternal integument might trigger the expression of 24-nt siRNAs in the embryo and initiate autonomous methylation in the embryo. This process would be analogous to the production of piRNAs in *Drosophila melanogaster*, whereby maternally derived small RNAs initiate subsequent filial siRNA production and transposon silencing [40]. It also remains possible that triggering siRNAs are brought to the zygote during fertilization by the sperm nucleus, although this model remains to be tested [41, 42].

Despite a lack of embryo hypermethylation, *rdr2* homozygous seeds from heterozygous mothers phenocopy wild-type seeds (Fig. 5b), indicating that embryo hypermethylation is not necessary for seed development in *B. rapa*. Similarly, *Arabidopsis* does not require DRM2 methyltransferase for embryo development [25]; however, *Arabidopsis* does not require RdDM generally, while other species in Brassicaceae have reproductive defects in the absence of RdDM [19]. Decoupling of embryo methylation and seed development in *B. rapa* supports the hypothesis that embryo hypermethylation is important for seed dormancy or longevity but not for seed development [22–24]. We assessed segregating seed populations and observed no difference in germination timing or frequency for unmethylated *rdr2* embryos relative to their methylated siblings (data not shown), suggesting that other hypotheses should also be considered.

In *Arabidopsis*, embryo hypermethylation is preferentially targeted to transposons in the pericentromeric heterochromatin [22, 23], while hypermethylation also occurs at euchromatic transposons in soybean [25, 27]. In *B. rapa*, we detect hypermethylation in both heterochromatin and euchromatin (Fig. 2b), suggesting that euchromatic embryo hypermethylation might be common among plants. Recent work demonstrates that *Arabidopsis* heterochromatin is decondensed and produces abundant 24-nt siRNAs during embryogenesis [43], providing an opportunity for the RdDM machinery to access this chromatin for hypermethylation.

Our demonstration that RdDM is responsible for the hypermethylation of embryos leads to the hypothesis that siRNAs would be abundant during embryogenesis. However, we were surprised by the low level of siRNAs at CHH hyper-DMWs in torpedo-stage embryos. Correlation between CHH levels in torpedo-stage and mature embryos (Fig. 1b) indicates that hypermethylation occurs throughout embryogenesis rather than during embryo maturation, and therefore, robust siRNA accumulation would be predicted. The 81,556 CHH hyper-DMWs account for ~10% of windows with sufficient read depth, and they accumulate 16.9% of the mapped siRNAs (20.1% of the mapped 24-nt siRNAs). While this is a substantial enrichment compared to the genome as a whole, these windows account for 13.8% of the mapped siRNAs (15.9% of the mapped 24-nt siRNAs) in leaves. This discrepancy suggests that while siRNA production is required for embryo hypermethylation, developmental-specific factors are required for robust methylation.

## Conclusions

*Brassica rapa* embryos are hypermethylated at both euchromatic and heterochromatic CHH positions. This hypermethylation requires RdDM, and there is no evidence that siRNAs from the endosperm or maternal somatic tissue direct embryo methylation. Successful development of seeds lacking embryo hypermethylation indicates that this methylation is not necessary for embryogenesis, even in species that require RdDM for seed development.

## Methods

### Plant materials and growth conditions

*Brassica rapa ssp trilocularis* variety R-o-18 were grown in a greenhouse at 70°/60°F (day/night) under at least 16 h of illumination. Plants were fully dried before seed collection. Dry seeds were soaked in water for no more than 60 min before manual dissection to remove mature embryos. Three wild-type or five *rdr2* mutant embryos were pooled before DNA extraction with the GeneJET Plant Genomic DNA Purification Kit (Thermo Fisher Scientific, K0791). Embryos from *rdr2/RDR2* heterozygous mothers were individually collected, prepped, and genotyped prior to DNA pooling. *rdr2* mutants were used in this study due to their complete loss of 24-nt siRNAs and strong developmental phenotype. Torpedo-stage endosperm and embryo samples were dissected from pistils that were manually pollinated with *B. rapa* genotype R500. Whole-genome bisulfite sequencing libraries were prepared as previously described [44]. Lambda Phage DNA (Promega D1521) was included as a bisulfite conversion control. Libraries were pooled and sequenced in a single lane of paired-end 76 nt on an Illumina NextSeq500 at the University of Arizona Genetics Core.

### Methylation analysis

Whole-genome bisulfite sequencing data from the ovule and leaf were obtained from NCBI (BioProject PRJNA588293, Additional file 1: Table S1). For other tissues, sequencing reads were quality controlled with FastQC [45, 46] and trimmed using Trim Galore (options --trim-n and --quality 20) [47]. Trimmed reads were aligned to *Brassica rapa* R-o-18 genome (v2.2, a kind gift from G.J. King and the *B. rapa* sequencing consortium) with bwameth [48]. To mark PCR duplicates and determine properly paired the alignment rate, Picard Tools [49] and Samtools [50] were respectively used, with options -q 10, -c, -F 3840, -f 66 for Samtools. We used Mosdepth [51] with option -x and -Q 10 and a custom Python script developed previously in the lab (bed_coverage_to_x_coverage.py, https://github.com/The-Mosher-Lab/grover_et_al_sirens_2020) to determine genomic coverage. Statistics for all libraries are found in Additional file 1: Table S1.

Percentage methylation per cytosine was extracted with MethylDackel [52] in two successive steps. The first step was to identify inclusion bounds based on methylation bias per read position using MethylDackel mbias, followed by MethylDackel extract. Since the default for MethylDackel is the CG context, we also used --CHG and --CHH options. We determined bisulfite conversion rates by alignment to the bacteriophage lambda (NCBI Genbank accession J02459.1) and *Brassica rapa* var. *pekinensis* chloroplast (NCBI Genbank accession NC 015139.1) genomes with a custom Python script developed previously in the lab (bedgraph_bisulfite_conv_calc.py, https://github.com/The-Mosher-Lab/grover_et_al_sirens_2020). Conversion frequencies were all above 99.4% (Additional file 1: Table S1). Replicates were checked for consistency by principal component analysis before pooling to increase read depth (Additional file 1: Fig. S6).

Methylation was calculated for each sample on 300-bp non-overlapping windows, which was made with the help of BEDTools makewindows [53] feature on the *Brassica rapa* R-o-18 genome. Pairwise methylation differences between tissues were measured using the methylKit package on merged alignment files for each tissue. We considered only those windows which had a *q* value of < 0.005 when calculating differentially methylated windows.

We calculated the enrichment of genomic features like small RNA loci, TEs, and genes within the CHH hyper-DMWs using BEDTools intersect [53, 54]. Transposable elements were annotated as in [31]. We considered features to be overlapping if there was at least 1 nucleotide shared. Genomic features were annotated onto the 300-bp non-overlapping windows using BEDTools makewindows [53], and the number of overlaps and non-overlaps between the hyper-DMWs and the genomic features were recorded. Fisher’s exact test was performed in R to determine if the number of overlaps indicated significant enrichment or depletion.

Methylation over pre-defined siren loci [31] was determined with BEDTools intersect [53] and a custom Python script (bedgraph_methylation_by_bed.py) developed previously [31] in the lab.

### Small RNA analysis

Small RNA sequencing datasets were obtained from NCBI (BioProject PRJNA588293, Additional file 1: Table S2). Small RNA processing (quality checking, non-coding RNA filtering, removal of reads mapping to chloroplast and mitochondrial genomes) was carried out with a publicly available small RNA data processing pipeline [55]. Only 19 to 26-nt reads were retained for further analysis. Replicates were pooled for better read alignment and depth. The genome was divided into 300-bp non-overlapping windows using BEDTools makewindows [53], and ShortStack [56, 57] was used to get read counts on each window (options --mismatches 0, --mmap u, --mincov 0.5 rpm, --pad 75 and --foldsize 1000). The sum of all 19-26nt small RNA reads from genomic windows or CHH hyper-DMWs were low and susceptible to count-based bias when normalized against total library size. Therefore, windows were binned into 5 sRNA expression levels and compared only within the same tissue.

## Supplementary Information

**Additional file 1.** : Supplementary Figure S1-6, Supplementary Table S1-3.

**Additional file 2.** Review history.

## Data Availability

The datasets supporting the conclusions of this article are available in the National Center for Biotechnology Information Sequence Read Archive under Bio Projects PRJNA588293 [58] and PRJNA657007 [59]. Small RNA datasets including leaf, embryo, and endosperm samples were obtained from Bio Project PRJNA588293 [58]. Whole-genome bisulfite datasets (leaf, ovule endosperm, torpedo embryo) were obtained from PRJNA588293 [58]. Whole-genome bisulfite datasets from mature embryo samples have been deposited to Bio Project PRJNA657007 [59].
