# Supplementary material for: Embryo CHH hypermethylation is mediated by RdDM and is autonomously directed in Brassica rapa
Source: Genome Biol. 2021 May 6;22:140. doi: 10.1186/s13059-021-02358-3 (PMC8101221; doi:10.1186/s13059-021-02358-3)
Supplement: Supplementary file 1 — Additional file 1. : Supplementary Figure S1-6, Supplementary Table S1-3. [file 13059_2021_2358_MOESM1_ESM.pdf]

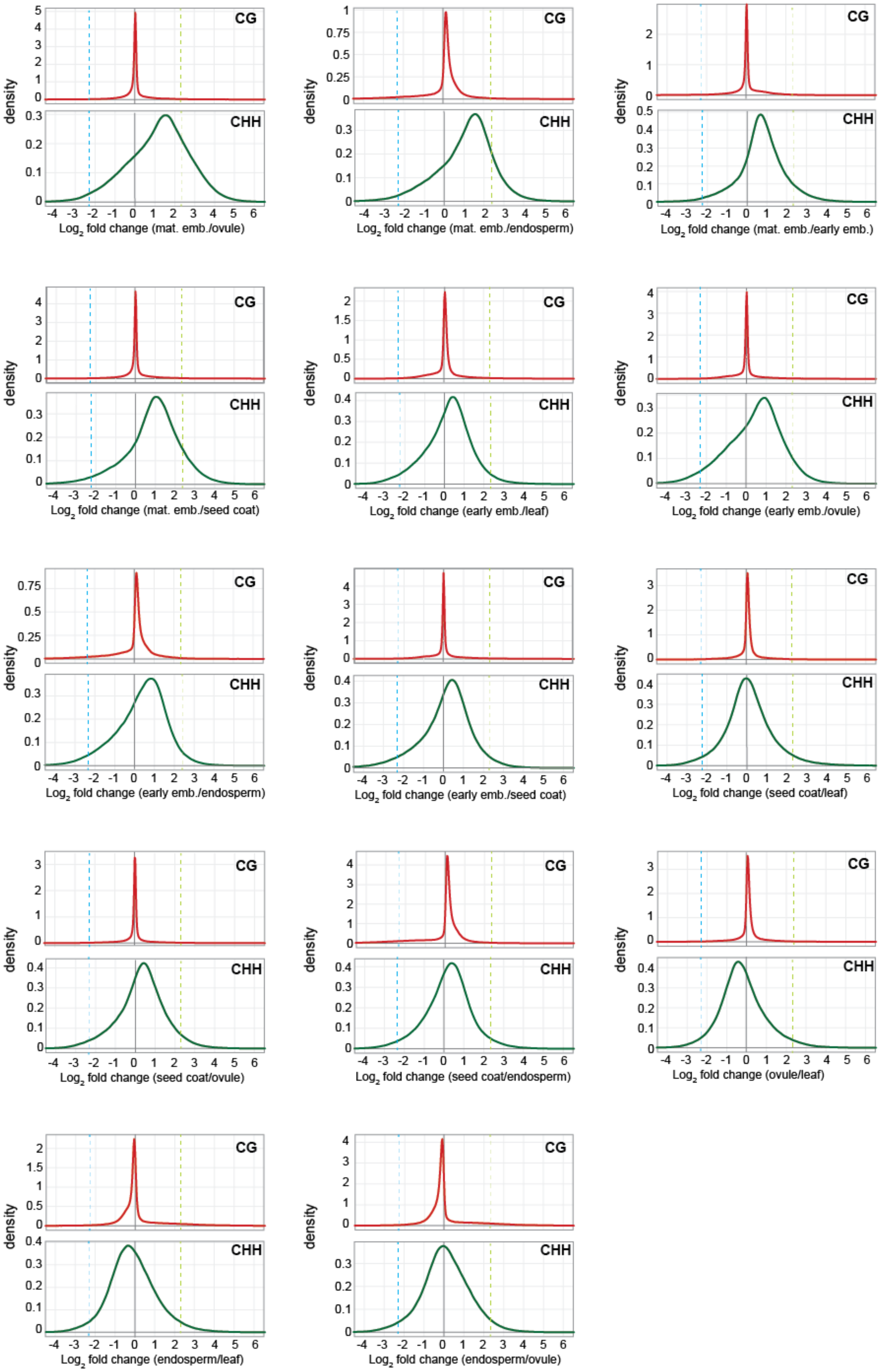

Supplemental Figure 1. CG and CHH distribution of methylation changes for all tissue comparisons. Density distributions plotted as in Figure 3.

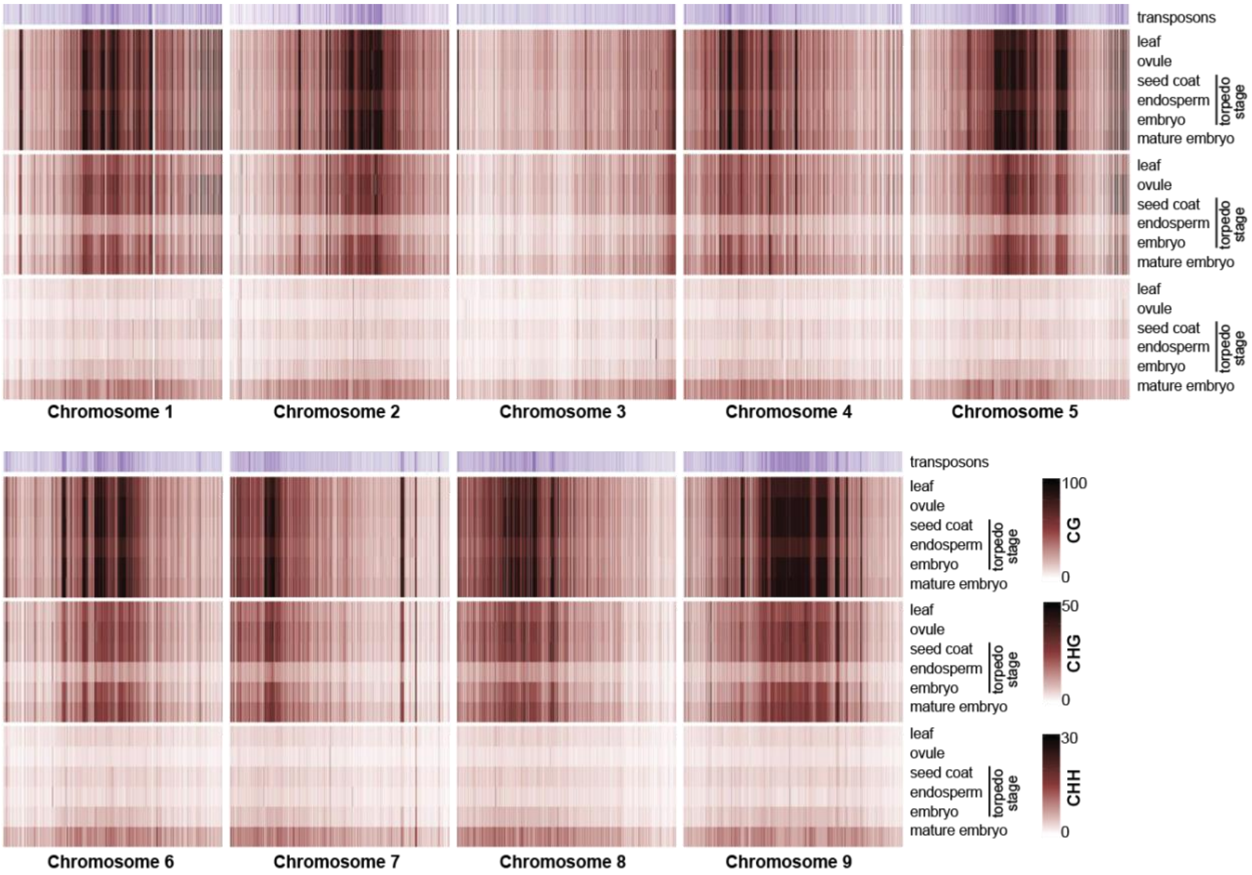

Supplemental Figure 2. Heatmaps of methylation on *B. rapa* chromosomes 1-9.
Heat maps of methylation in 25-kb windows across *B. rapa* chromosomes, as in Figure 2A.

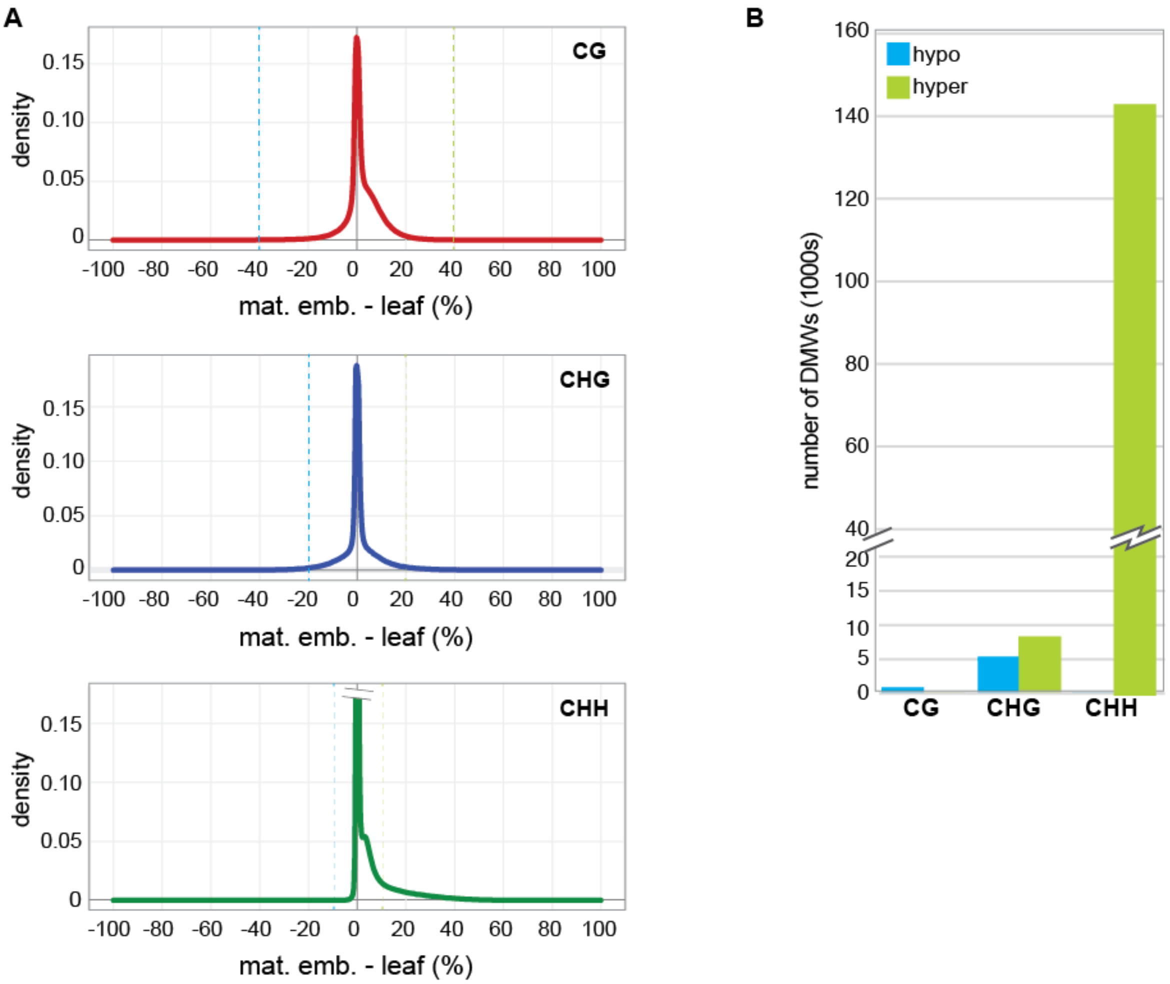

Supplemental Figure 3. Alternative method for assessing changes in DNA methylation.
(A) The distribution of difference in methylation (mature embryo % methylation – leaf % methylation) at 300-nt genomic windows with at least 5 reads coverage. Dashed yellow and blue lines denote the difference thresholds used to identify substantial changes in methylation (40% for CG, 20% for CHG, and 10% for CHH). The number of windows beyond these thresholds and having an FDR-adjusted p-value < 0.005 are shown in (B). Similar to the fold-change calculation in Figure 3, this alternative calculation demonstrates that the primary difference in methylation between leaves and mature embryos is hypermethylation of CHH sites.

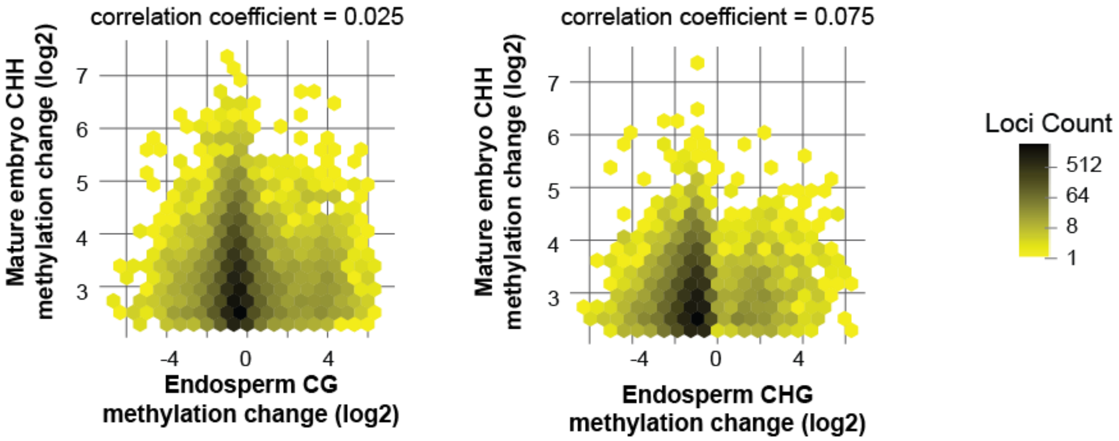

Supplemental Figure 4. No correlation between endosperm demethylation and embryo hypermethylation at CHH hyper-DMWs.

Hex plots of mature embryo CHH methylation change by torpedo-stage endosperm CG (left) or CHG (right) methylation change. Only CHH hyper-DMWs are plotted (whole genome plotted in Figure 5C).

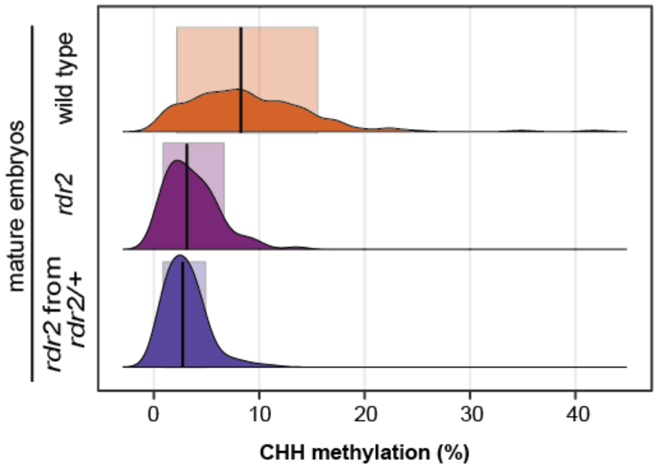

Supplemental Figure 5. Mature embryo methylation at siren loci.
Distribution of CHH methylation at siren loci in mature embryos. *rdr2* embryos were derived either from *rdr2* homozygous mothers (maroon) or from *rdr2/+* heterozygous mothers (purple).

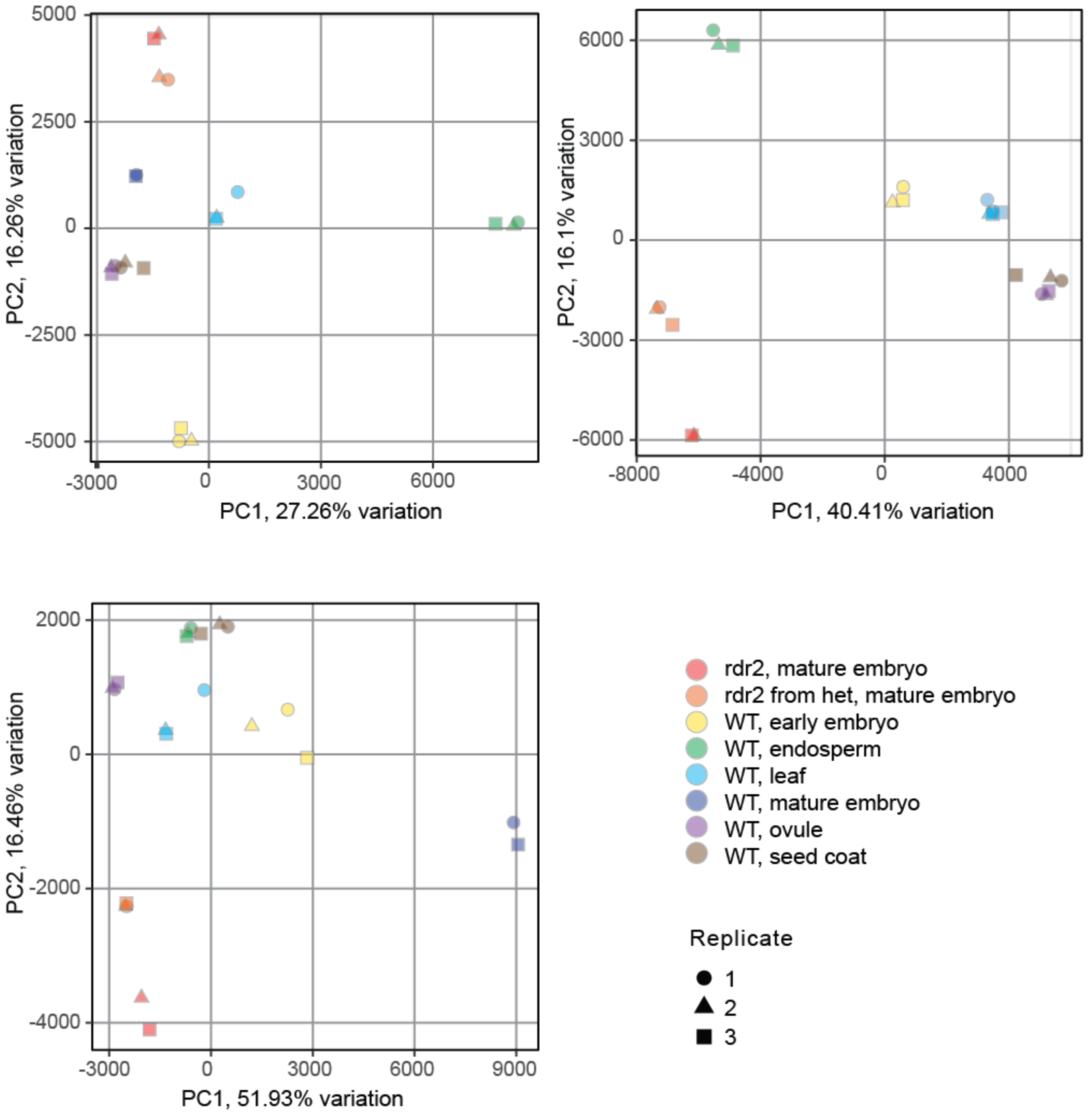

Supplemental Figure 6. PCA analysis of WGBS replicates

Supplemental Table 1. WGBS sequence datasets

Genotype	Tissue	Replicate	NCBI identifier	De-multiplexed Reads	Deduplicated Alignments	Mapping Rate %	Coverage X	Conversion Rate (lambda DNA)	Conversion Rate (chloroplast DNA)
WT R-o-18	leaf	1	SRX7113698	33,843,130	18,626,017	55.43%	8.24	99.66	99.60
WT R-o-18	leaf	2	SRX7113699	34,414,305	16,928,242	49.56%	7.46	99.68	99.66
WT R-o-18	leaf	3	SRX7113700	39,216,330	19,653,280	50.69%	8.73	99.63	99.61
WT R-o-18	ovule	1	SRX7113704	35,677,253	20,580,397	58.35%	9.08	99.64	99.52
WT R-o-18	ovule	2	SRX7113705	38,415,932	22,250,022	58.58%	9.81	99.65	99.51
WT R-o-18	ovule	3	SRX7113706	33,159,726	19,047,779	58.06%	8.38	99.63	99.47
WT R-o-18 x R500	endosperm	1	SRX8941582	37,703,830	20,112,987	54.30%	9.09	99.71	99.55
WT R-o-18 x R500	endosperm	2	SRX8941583	40,064,286	20,957,711	53.18%	9.61	99.69	99.49
WT R-o-18 x R500	endosperm	3	SRX8941584	42,843,140	22,831,013	53.88%	10.40	99.70	99.50
WT R-o-18 x R500	seed coat	1	SRX8941585	37,809,229	20,014,869	53.89%	9.10	99.71	99.36
WT R-o-18 x R500	seed coat	2	SRX8941586	38,519,379	19,894,934	50.06%	8.94	99.76	99.43
WT R-o-18 x R500	seed coat	3	SRX8941587	40,731,277	21,863,906	54.61%	9.95	99.69	99.36
WT R-o-18 x R500	torpedo embryo	1	SRX8941594	37,095,900	18,335,536	49.81%	5.81	99.72	99.65
WT R-o-18 x R500	torpedo embryo	2	SRX8941595	37,168,809	18,136,734	50.34%	8.35	99.71	99.66
WT R-o-18 x R500	torpedo embryo	3	SRX8941581	39,057,700	19,063,283	49.35%	8.94	99.67	99.63
rdr2 R-o-18	mature embryo	1	SRX8941580	42,990,881	22,738,273	52.89%	10.02	99.57	99.56
rdr2 R-o-18	mature embryo	2	SRX8941588	44,373,420	24,006,617	54.10%	10.51	99.52	99.48
rdr2 from het mother R-o-18	mature embryo	1	SRX8941589	48,591,584	24,052,250	49.50%	10.83	99.62	99.57
rdr2 from het mother R-o-18	mature embryo	2	SRX8941590	45,727,329	22,713,974	49.67%	10.11	99.56	99.54
rdr2 from het mother R-o-18	mature embryo	3	SRX8941591	45,076,722	21,617,299	47.96%	9.77	99.59	99.56
WT R-o-18	mature embryo	1	SRX8941592	48,525,785	24,826,190	51.16%	10.95	99.43	99.52
WT R-o-18	mature embryo	2	SRX8941593	40,351,733	20,545,946	50.92%	9.04	99.50	99.57

Supplemental Table 2. sRNA sequence datasets

Genotype	Tissue	Replicate	NCBI identifier	De-multiplexed Reads	Trimming & q > 30 filtering	% Remaining	rfam *B. rapa* & *A. thaliana* Filtered	% Remaining	*B. rapa* and *A. thaliana* CM Filtered	% Remaining	Mapped to R-o-18 and 19-26nt Small RNAs filtered	% Remaining
WT R-o-18	17dpf endosperm	1	SRX7113618	14,782,390	6,319,548	42.75%	6,319,238	99.9951%	6,210,368	98.277%	4,033,404	64.95%
WT R-o-18	17dpf endosperm	2	SRX7113619	23,412,918	9,108,385	38.90%	9,107,867	99.9943%	8,963,673	98.417%	6,033,604	67.31%
WT R-o-18	17dpf endosperm	3	SRX7113620	14,579,403	5,827,576	39.97%	5,827,156	99.9928%	5,718,292	98.132%	3,618,539	63.28%
WT R-o-18	17dpf embryo	1	SRX7113621	21,823,191	15,937,453	73.03%	15,936,988	99.9971%	15,445,456	96.916%	10,619,292	68.75%
WT R-o-18	17dpf embryo	2	SRX7113622	26,629,773	18,404,570	69.11%	18,404,128	99.9976%	17,914,651	97.340%	12,031,834	67.16%
WT R-o-18	17dpf embryo	3	SRX7113623	21,903,128	13,863,523	63.29%	13,863,171	99.9975%	13,459,146	97.086%	9,174,737	68.17%
WT R-o-18	Leaves	1	SRX7113689	27,441,990	10,767,807	39.24%	10,767,489	99.9970%	8,391,872	77.937%	3,635,851	43.33%
WT R-o-18	Leaves	2	SRX7113690	33,992,611	11,177,367	32.88%	11,176,716	99.9942%	9,002,798	80.550%	4,033,874	44.81%
WT R-o-18	Leaves	3	SRX7113691	29,436,807	9,381,536	31.87%	9,381,375	99.9983%	7,546,841	80.445%	3,146,617	41.69%
WT R-o-18xR-o-18	Embryo	1	SRX7113624	21,304,038	4,941,922	23.20%	4,941,786	99.9972%	4,804,648	97.225%	3,098,803	64.50%
WT R-o-18xR-o-18	Embryo	2	SRX7113625	16,203,619	4,132,008	25.50%	4,131,841	99.9960%	4,014,630	97.163%	2,726,752	67.92%
WT R-o-18xR-o-18	Embryo	3	SRX7113626	18,581,017	2,302,004	12.39%	2,301,886	99.9949%	2,199,864	95.568%	1,446,836	65.77%

Supplemental Table 3. Pearson correlation coefficients, r, for endosperm to embryo methylation.

Correlation between endosperm methylation and mature embryo CHH methylation at all genomic windows with sufficient read depth

	absolute methylation (%)	fold change	difference in methylation (%)
CG	0.53	-0.19	-0.36
CHG	0.69	-0.093	-0.48
CHH	0.82	0.38	0.19

Correlation between endosperm methylation and mature embryo CHH methylation at CHH hyper-DMWs

	absolute methylation (%)	fold change	difference in methylation (%)
CG	0.37	0.025	-0.23
CHG	0.45	0.075	-0.35
CHH	0.60	0.51	0.34
