# Supplementary material for: Embryo CHH hypermethylation is mediated by RdDM and is autonomously directed in Brassica rapa
Source: Genome Biol. 2021 May 6;22:140. doi: 10.1186/s13059-021-02358-3 (PMC8101221; doi:10.1186/s13059-021-02358-3)
Supplement: Supplementary file 2 — Additional file 2. Review history. [file 13059_2021_2358_MOESM2_ESM.docx]

Review History

**First round of review**

**Reviewer 1**

**Are you able to assess all statistics in the manuscript, including the appropriateness of statistical tests used?**

There are no statistics in the manuscript.

**Comments to author:**

Chakraborty and colleagues examined DNA methylation in late stage Brassica rapa embryos, compared this with published small RNA sequencing datasets, and found that CHH methylation is mediated by RdDM in Brassica rapa embryos. They also performed a clever experiment by crossing rdr2/+ mothers with rdr2 fathers followed by methylation analysis of rdr2/rdr2 embryos from rdr2/+ mothers and based on these results concluded that small RNAs produced in maternal sporophytic tissues are not sufficient for the hypermethylation observed in mature embryos. By examining methylation in endosperm and small RNA levels in embryos, they also did not find evidence that hypomethylated regions of the endosperm genome initiates the production of small RNAs that are then transported into the embryo. These results in Brassica rapa support previous conclusions from studies with Arabidopsis thaliana embryos.

Progressive CHH hypermethylation in Arabidopsis embryos was reported by Bouyer et al., Kawakatsu et al. and Pappareddy et al. The Kawakatsu and Pappareddy publications also showed that RdDM and CMT2 pathways mediate this hypermethylation in pericentromeric and chromosomal arms and provide evidence that siRNAs are autonomously produced in embryos already at early stages. Therefore, the results in the current manuscript agree with previous publications. Erdmann et al. Cell Reports (which was not cited in this manuscript) also found that equal amounts of 24-nt small RNAs are produced by maternal and paternal chromosomes in Arabidopsis torpedo embryos indicating that small RNAs are autonomously produced in late stage embryos. Therefore, the conclusions of Chakraborty et al. in Brassica rapa have already been demonstrated in Arabidopsis, which is another member of the Brassicaceae family.

My major criticism is that although this is an interesting study in an agriculturally important plant, it confirms what has already been shown in Arabidopsis.

Minor criticisms:

I did not understand the title/conclusion of Figure 4. How do the authors conclude that small RNA production, but not abundance, is correlated with embryo hypermethylation?

The authors should also elaborate on how previous correlations between endosperm demethylation and embryo hypermethylation are "artifactual".

The relevance of the last paragraph of the Discussion (starting on line 253) was also not clear to me.

**Reviewer 2**

**Are you able to assess all statistics in the manuscript, including the appropriateness of statistical tests used?**

Yes, and I have assessed the statistics in my report.

**Comments to author:**

In Chakraborty et al, Mosher and colleagues continue their investigation of the role of RdDM in seed development in B. rapa. This series of studies have been useful in demonstrating how RdDM has diverged even among fairly closely related species. In this study, they probe the role of RdDM in embryo CHH hypermethylation. This is a good addition to multiple studies that are probing DNA methylation at different developmental stages including during seed development. It shows that canonical RdDM is needed in the mother but not in the embryo for seed viability. This paper also provides evidence against the model that small RNAs move from endosperm to embryo- a model which has gained a lot of traction without much/any evidence. I recommend that this paper be published. However, I suggest that the authors consider the following comments.

Minor comments

1.     While the overall conclusions are unlikely to change and I do not wish that the authors redo the analysis for the paper itself, I feel that the authors, by using non-overlapping windows, may have missed informative changes in methylation that can in the future help identify DNA methylation controlling gene expression.

2.     Authors use Figure 1A to conclude that CG methylation was largely unchanged. However, while the quantity of CG methylation, genome-wide, may not have changed, the exact location may have changed. I suggest that the authors calculate differences in methylation between windows to draw such a conclusion.

3.     In Figure 1B, having the same scales on X and Y axes will make interpretation easy for the reader.

4.     In Figure 3, sites without CHH methylation in  leaf (0% CHH) may have CHH methylation in embryo. How many sites are being missed in this graph since you are looking at Log2(embryo/leaf) and sites where the denominator is zero are going to be likely missed. Would a better way to present this data be % methylation (emb-leaf)?

5.     In Line 119, the authors state " Together, our observations demonstrate that mature B. rapa embryos are extensively hypermethylated at CHH sites across the genome, and this hypermethylation is the primary difference  between leaf and embryo methylation patterns."

The authors should do more to back up this claim. They should have a graph which  tells the reader how many sites have gained methylation and by how much. The log(fold change) is not a very informative statistic because methylation can change from 0.1 to 0.8% and that ratio is the same as changing from 10 to 80%. The latter is actually hypermethylated while the former is the embryo being hypermethylated relative to the leaf.

6.     Are the genes with CHH hyper DMRs in the embryo evolutionarily young genes.

7.     The authors should note why they chose rdr2 mutants over nrpd1 or nrpe1?

8.     In regions that have rdr2 dependent DNA methylation, is there a relationship with number of sRNAs and DNA methylation? Are there regions which are likely to have both RDR2 dependent and independent CHH methylation?

9.     Is there still DRM2 dependent DNA methylation that is laid down in RDR2 mutants, perhaps via RDR6? Have the authors thought of using the kind of analysis detailed in Harris and Zemach (2020) to delineate DRM2 and CMT2 dependent DNA methylation and see if DRM2 dependent DNA methylation is still functional?

10.     Are there sites within the embryo whose DNA methylation levels show dose dependent changes to RDR2?

**Authors Response**

**Point-by-point responses to the reviewers’ comments:**

Reviewer #1:

Chakraborty and colleagues examined DNA methylation in late stage Brassica rapa embryos, compared this with published small RNA sequencing datasets, and found that CHH methylation is mediated by RdDM in Brassica rapa embryos.

They also performed a clever experiment by crossing rdr2/+ mothers with rdr2 fathers followed by methylation analysis of rdr2/rdr2 embryos from rdr2/+ mothers and based on these results concluded that small RNAs produced in maternal sporophytic tissues are not sufficient for the hypermethylation observed in mature embryos.

By examining methylation in endosperm and small RNA levels in embryos, they also did not find evidence that hypomethylated regions of the endosperm genome initiates the production of small RNAs that are then transported into the embryo. These results in Brassica rapa support previous conclusions from studies with Arabidopsis thaliana embryos.

Progressive CHH hypermethylation in Arabidopsis embryos was reported by Bouyer et al., Kawakatsu et al. and Pappareddy et al. The Kawakatsu and Pappareddy publications also showed that RdDM and CMT2 pathways mediate this hypermethylation in pericentromeric and chromosomal arms and provide evidence that siRNAs are autonomously produced in embryos already at early stages. Therefore, the results in the current manuscript agree with previous publications.

Erdmann et al. Cell Reports (which was not cited in this manuscript) also found that equal amounts of 24-nt small RNAs are produced by maternal and paternal chromosomes in Arabidopsis torpedo embryos indicating that small RNAs are autonomously produced in late stage embryos. Therefore, the conclusions of Chakraborty et al. in Brassica rapa have already been demonstrated in Arabidopsis, which is another member of the Brassicaceae family.

My major criticism is that although this is an interesting study in an agriculturally important plant, it confirms what has already been shown in Arabidopsis.

*Yes, our results agree with these publications, and we believe our manuscript extends them in important ways. While Kawakatsu et al. conclude that “hypermethylation during seed development relied on both RdDM and CMT2 pathways”, they did not directly test RdDM mutants but rather triple and quadruple methyltransferase mutants. They also analyzed TEs pre-defined as “RdDM-targeted” or “CMT2-targeted”, however those definitions were based on leaves rather that developing embryos, and therefore might not represent the regulation of those TEs during embryogenesis. Papareddy et al. did test a mutant lacking the RdDM pathway and concluded that RdDM is responsible for much of the CHH hypermethylation detected during embryogenesis, however they make no conclusions regarding maternal or zygotic control of this pathway. (It is also worth noting that while we were aware of Papareddy et al. due to a preprint on BioRxiv, the peer-reviewed version was not published at the time we submitted this manuscript (Sept 4, 2020)).*

*Finally, while Erdmann et al. detect biparental siRNA populations in early-stage embryos, which suggests autonomous production, they also detect regions that are parentally-biased, and their manuscript makes no conclusion regarding autonomous or non-autonomous production. Nevertheless, this is an important reference to include and we have added it to the manuscript (lines 71-72).*

*Most importantly, we believe that “confirm[ing] what has already been shown in Arabidopsis” is critically important in this case. RdDM is not required for seed development in Arabidopsis, and only impacts reproduction under very specific and uncommon conditions. Brassica rapa, which requires maternal sporophytic RdDM for seed development, is therefore more representative of flowering plants. (Tomato, rice, and maize also have defective reproduction when RdDM is eliminated.) Because B. rapa is a close relative of Arabidopsis, yet differs in the role of RdDM during seed development, comparisons of the two species are critical. By demonstrating that embryo hypermethylation is an area in which the two species are similar, we have eliminated the possibility that differences in embryo hypermethylation lead to different requirements for RdDM during reproduction.*

Minor criticisms:

I did not understand the title/conclusion of Figure 4. How do the authors conclude that small RNA production, but not abundance, is correlated with embryo hypermethylation?

*Thank you for pointing out this lack of clarity. We observed that within embryos there is a correlation between siRNA accumulation and hypermethylation, however this correlation does not extend to leaf samples, which also show elevated siRNA accumulation but lack hypermethylation. We have reworded the figure title and added additional description (lines 148-150).*

The authors should also elaborate on how previous correlations between endosperm demethylation and embryo hypermethylation are "artifactual".

*We have edited this conclusion to be more specific. Lines 182-184 now read “these results suggest that while similar loci may be demethylated in endosperm and hypermethylated in embryos, there is no evidence that demethylation of endosperm causes hypermethylation of embryos in B. rapa”.*

The relevance of the last paragraph of the Discussion (starting on line 253) was also not clear to me.

*We have added a sentence at the beginning of this paragraph to clarify our logic (lines 271-272). Specifically, because RdDM is responsible for hypermethylation of embryos, one might expect that embryos would accumulate abundant siRNAs from the hypermethylated regions during embryogenesis. However, we did not observe this. Instead, hyper-DMWs account for approximately the same fraction of siRNAs in embryos as in leaves, despite the difference in methylation of these tissues. We therefore propose that there are developmental-specific factors that influence the efficacy of RdDM during embryogenesis.*

Reviewer #2:

In Chakraborty et al, Mosher and colleagues continue their investigation of the role of RdDM in seed development in B. rapa. This series of studies have been useful in demonstrating how RdDM has diverged even among fairly closely related species. In this study, they probe the role of RdDM in embryo CHH hypermethylation. This is a good addition to multiple studies that are probing DNA methylation at different developmental stages including during seed development.

It shows that canonical RdDM is needed in the mother but not in the embryo for seed viability. This paper also provides evidence against the model that small RNAs move from endosperm to embryo- a model which has gained a lot of traction without much/any evidence. I recommend that this paper be published.

*Thank you for your support and for the helpful suggestions below.*

However, I suggest that the authors consider the following comments.

Minor comments

1.      While the overall conclusions are unlikely to change and I do not wish that the authors redo the analysis for the paper itself, I feel that the authors, by using non-overlapping windows, may have missed informative changes in methylation that can in the future help identify DNA methylation controlling gene expression.

*The reviewer points to a long-standing concern in how to analyze differentially methylated regions. While non-overlapping regions might have missed smaller regions that were unlucky and split between windows, overlapping windows counts any region that is not split in this way twice. Given our focus on hypermethylated regions as a whole, rather than identifying specific DMRs associated with genes, we preferred to avoid this redundancy. We encourage anyone hoping to identify DNA methylation controlling gene expression to reanalyze our data in a way that is most appropriate to their question, and we have deposited our data in the NCBI SRA for this purpose.*

2.      Authors use Figure 1A to conclude that CG methylation was largely unchanged. However, while the quantity of CG methylation, genome-wide, may not have changed, the exact location may have changed. I suggest that the authors calculate differences in methylation between windows to draw such a conclusion.

*To assess whether the global distribution of CG methylation is unchanged due to balanced gain and loss of methylation in different locations, we calculated changes in CG methylation between leaf and all other tissues. Density plots of these differences are now found in Supplemental Figure 1. This analysis supports our conclusion that there is little change in CG methylation between these tissues, which is in marked contrast to the change in CHH methylation, including hypermethylation in both early and mature embryos.*

3.      In Figure 1B, having the same scales on X and Y axes will make interpretation easy for the reader.

*Thank you for this recommendation. We have updated the figure panel as suggested.*

4.      In Figure 3, sites without CHH methylation in leaf (0% CHH) may have CHH methylation in embryo. How many sites are being missed in this graph since you are looking at Log2(embryo/leaf) and sites where the denominator is zero are going to be likely missed. Would a better way to present this data be % methylation (emb-leaf)?

*Although 1097 windows are not plotted on this graph because they have no methylation in leaves, these windows were included in our later analysis hyper-DMWs because they represent increased CHH methylation in embryos.*

*Much like the decision regarding overlapping or non-overlapping windows, we had to decide which method to use to identify differentially methylated regions. We prefer to use fold change rather than subtraction, but for some analyses (e.g., Supplemental Table 3) we compare fold change and difference in methylation. For readers who prefer to see subtraction, we have replicated Figures 3A and 3C using the subtraction method and placed these as Supplemental Figure 3.*

5.      In Line 119, the authors state "Together, our observations demonstrate that mature B. rapa embryos are extensively hypermethylated at CHH sites across the genome, and this hypermethylation is the primary difference  between leaf and embryo methylation patterns."

The authors should do more to back up this claim. They should have a graph which tells the reader how many sites have gained methylation and by how much. The log(fold change) is not a very informative statistic because methylation can change from 0.1 to 0.8% and that ratio is the same as changing from 10 to 80%. The latter is actually hypermethylated while the former is the embryo being hypermethylated relative to the leaf.

*While the reviewer might prefer to subtract methylation levels, this metric is also not perfect as it treats a window with a substantial change (eg, 1-21% methylation) the same as a window a minimal change (eg, 80-100%). Unfortunately, both methods have their weaknesses. It is true that fold-change can be susceptible to noisy data in the low range (the 0.1 to 0.8% methylation change mentioned). We addressed this issue by using biological triplicates and only selecting windows with an FDR-corrected p-value less than 0.005. So while some of the changes we detect might be small in absolute terms, they are reproducible and therefore represent hypermethylation of the embryo relative to the leaf.*

*Nevertheless, as described in point 4 above, we have now added Supplemental Figure 4, which represents the distribution of methylation difference between the embryo and leaf in each cytosine context. This figure supports our conclusion that the change in methylation from leaves to embryos is primarily hypermethylation of CHH sites. The secondary method returns a greater number of windows (16% of all analyzed CHH windows), and these windows substantially overlap with those we analyze (see below), suggesting that it may be a less stringent measure than the one we prefer but one that returns largely similar windows.*

6.      Are the genes with CHH hyper DMRs in the embryo evolutionarily young genes?

*We are not sure what the reviewer means in this question. All of the genes in the B. rapa genome were subject to a whole genome triplication 10-30 million years ago. Many have returned to single copy, while at least some of the others have subfunctionalized or neofunctionalized. Determining which genes retain their ancestral function or have evolved since their duplication is beyond the scope of this manuscript.*

7.      The authors should note why they chose rdr2 mutants over nrpd1 or nrpe1?

*We have now included a justification for using rdr2 in the methods section (lines 300-301). Of the three rddm mutants that we have assessed in Brassica rapa, rdr2 has a strong developmental phenotype as well as near complete loss of 24-nt siRNAs. Although it would be interesting to determine whether nrpe1 is required for embryo hypermethylation, because this mutant produces 24-nt siRNAs, we would not be able to test whether maternally-derived siRNAs could contribute to embryo hypermethylation. Our nrpd1 allele is a missense mutation rather than a nonsense mutation and small RNA sequencing suggests that it might retain a small amount of activity. Since we only had resources to focus on one mutant, we therefore selected rdr2.*

8.      In regions that have rdr2 dependent DNA methylation, is there a relationship with number of sRNAs and DNA methylation? Are there regions which are likely to have both RDR2 dependent and independent CHH methylation?

*Since rdr2 returns the embryo to leaf-like methylation levels (Figure 5A), regions with RDR2-dependent methylation are largely similar to the hyper-DMWs assessed earlier in the manuscript. Figure 4C shows that hyper-DMWs with more siRNAs in torpedo embryos become more methylated in mature embryos, suggesting that there is a relationship between number of siRNAs and DNA methylation. However, leaves also have high siRNA production from these regions, so siRNA production is not the sole determinant of methylation level.*

*To assess the role of RDR2-dependent versus RDR-independent methylation in embryo hypermethylation, we measured how much WT methylation is lost in rdr2 ((rdr2 embryo – WT embryo)/WT embryo). This is now figure panel 5C and shows that 40% of embryo hyper-DMWs lose at least three-quarters (75%) of their CHH methylation in rdr2. Another ~40% of embryo hyper-DMWs retain 25-50% of the WT methylation (50-75% lost in rdr2), suggesting that RDR2-independent methylation plays a small role at these loci. This idea is now discussed in lines 155-157.*

9.      Is there still DRM2 dependent DNA methylation that is laid down in RDR2 mutants, perhaps via RDR6? Have the authors thought of using the kind of analysis detailed in Harris and Zemach (2020) to delineate DRM2 and CMT2 dependent DNA methylation and see if DRM2 dependent DNA methylation is still functional?

*We have not yet attempted to distinguish between DRM2 and CMT2-mediated methylation in Brassica, mainly because we do not have the necessary mutants to discern the methylation of these individual pathways. We agree that exploring these questions beyond Arabidopsis is an interesting research area, particularly for species with developmental phenotypes associated with DNA methylation pathways.*

10.     Are there sites within the embryo whose DNA methylation levels show dose dependent changes to RDR2?

*Unfortunately, we do not have the datasets to address this question, as we did not sequence methylomes from rdr2/RDR2 heterozygotes, but only from rdr2 homozygous embryos with heterozygous mothers.*

**Second round of review**

**Reviewer 2**

I have no further comments.
